# Elucidation of Transport Mechanism of Paeoniflorin and the Influence of Ligustilide, Senkyunolide I and Senkyunolide A on Paeoniflorin Transport through Mdck-Mdr1 Cells as Blood–Brain Barrier *in Vitro* Model

**DOI:** 10.3390/molecules21030300

**Published:** 2016-03-02

**Authors:** Peng-Yi Hu, Dan Liu, Qin Zheng, Qing Wu, Yu Tang, Ming Yang

**Affiliations:** 1 School of Chinese Materia Medica, Beijing University of Chinese Medicine, Beijing 100102, China; hpy820515@126.com; 2 Key Laboratory of Modern Preparation of Traditional Chinese Medicine, Ministry of Education, Jiangxi University of Traditional Chinese Medicine, Nanchang 330004, China; 18070142107@163.com (D.L.); ty505783@163.com (Y.T.); yangming16@126.com (M.Y.)

**Keywords:** paeoniflorin, ligustilide, senkyunolide I, senkyunolide A, blood–brain barrier, P-gp

## Abstract

The objectives of the present investigation were to: (1) elucidate the transport mechanism of paeoniflorin (PF) across MDCK-MDR1 monolayers; and (2) evaluate the effect of ligustilide (LIG), senkyunolide I (SENI) and senkyunolide A (SENA) on the transport of PF through blood–brain barrier so as to explore the enhancement mechanism. Transport studies of PF were performed in both directions, from apical to basolateral side (A→B) and from basolateral to apical sides (B→A). Drug concentrations were analyzed by LC-MS/MS. PF showed relatively poor absorption in MDCK-MDR1 cells, apparent permeability coefficients (Papp) ranging from 0.587 × 10^−6^ to 0.705 × 10^−6^ cm/s. *In vitro* experiments showed that the transport of PF in both directions was concentration dependent and not saturable. The B→A/A→B permeability *ER* of PF was more than 2 in the MDCK-MDR1 cells, which indicated that the transport mechanism of PF might be passive diffusion as the dominating process with the active transportation mediated mechanism involved. The increased Papp of PF in A→B direction by EDTA-Na_2_ suggested that PF was absorbed via the paracellular route. The P-gp inhibitor verapamil could significantly increase the transport of PF in A→B direction, and *ER* decreased from 2.210 to 0.690, which indicated that PF was P-gp substance. The transport of PF in A→B direction significantly increased when co-administrated with increasing concentrations of LIG, SENI and SENA. An increased cellular accumulation of Rho 123 and Western blot analysis indicated that LIG, SENI and SENA had increased the transport of PF in the BBB models attribute to down-regulate P-gp expression. A decrease in transepithelial electrical resistance (TEER) during the permeation experiment can be explained by the modulation and opening of the tight junctions caused by the permeation enhancer LIG, SENI and SENA.

## 1. Introduction

**Figure 1:**
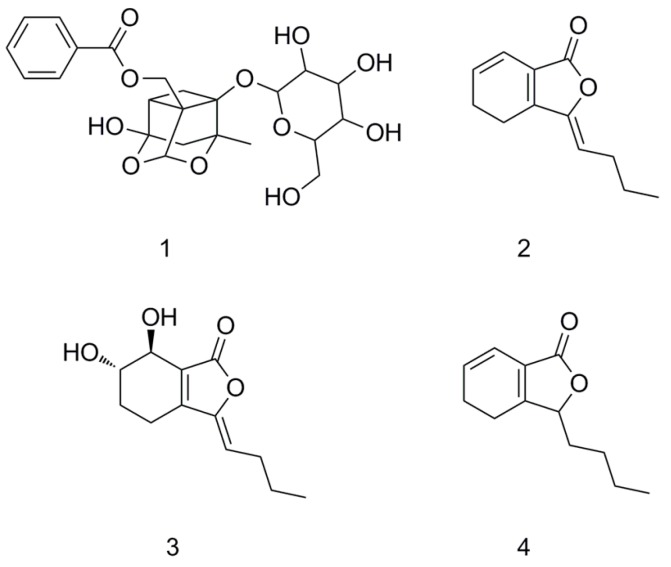
Chemical structure of paeoniflorin (**1**), ligustilide (**2**), senkyunolide I (**3**) and senkyunolide A (**4**).

Paeoniflorin (PF, Figure 1(**1**)), a monoterpene glycoside, is one of the principal bioactive components extracted from the root of *Paeonia lactiflora* pall that has been used in Traditional Chinese Medicine for thousands of years [1,2]. Recently, the effect PF has on the nervous system has attracted more attention. PF can ameliorate the decline of memory and learning capacity, protect the cerebral ischemia injury, have effects on the treatment of Alzheimer’s disease, relieve pain and improve neural synapse plasticity [3,4,5]. PF is a highly water-soluble phenolic glucoside with poor liposolubility [6,7], and does not easily pass through the blood–brain barrier (BBB). Therefore, enhancing the delivery of PF to the brain could improve therapeutic efficacy.

In many prescriptions of Traditional Chinese Medicine (TCM), certain TCM, called messenger drugs, are considered capable of introducing the main effective drugs in the prescription to the target site to increase therapeutic efficacy. Chuanxiong (*Ligusticum chuanxiong* Hort.) is one such messenger drug, which is frequently used in the treatment of encephalopathy [8]. Our previous research and some reports have shown that it could enhance drug absorption through gastrointestinal tract [9], and increase the distribution of drugs in brain tissue [10,11,12]. The combination of chuanxiong and PF is always used to treat CNS diseases. Traditional Chinese herbal formula Danggui Shaoyao San, which contains *Paeonia lactiflora* Pall., *Ligusticum chuanxiong* Hort., *Angelica sinensis* (Oliv.) Diels., *Poria cocos* (Schw.) Wolf, *Alisma orientale* (Sam.) Juzep. and *Atractylodes macrocephala* Koidz., has been used to treat ischemic stroke and Alzheimer’s diseases [13,14]. PF was one of the main active components in Danggui-Shaoyao-San. Another Traditional Chinese herbal formula Buyang Huanwu decoction, which has been commonly used for the treatment of stroke, also contains chuanxiong and PF [15]. Buyang Huanwu decoction containing chuanxiong has a better therapeutic effecton cerebral is chemia than Buyang Huanwu decoction without chuanxiong, which further confirmed that chuanxiong was a messenger drug. However, the promoting ingredients in chuanxiong and the mechanism are not yet known.

Chuanxiong contains phenolic acids, lactones, alkaloids, volatile oil and other ingredients [16]. Volatile oil of chuanxiong could enhance drug permeation through skin [17], promote the survival of cerebral cortex neurons *in vitro*, and alleviate the damages caused by ischemia reperfusion [18]. Ligustilide (Figure 1(**2**), LIG), Senkyunolide I (Figure 1(**3**), SENI) and Senkyunolide A (Figure 1(**4**), SENA), phthalide compounds in volatile oil, have various pharmacological effects closely associated with chuanxiong [19,20,21] and could be absorbed into rat brain tissues through BBB [22]. In this article, LIG, SENI and SENA were regarded as promoting ingredients of chuanxiong to study the underlying mechanisms.

P-glycoprotein (P-gp), encoded by the ABCB1 gene, belongs to the ATP-binding cassette superfamily and is constitutively expressed at the luminal membrane of the brain endothelial cells [23]. The biological function of P-gp is to protect brain from toxic xenobiotics by the energy-dependent efflux of substrates. However, it causes the intracellular concentrations of drugs to decrease and may result in treatment failure. Thus, compounds that inhibit P-gp activities may have the potential to improve the clinical outcomes of drugs [24]. Until now, it was unclear whether LIG, SENA and SENI could inhibit P-gp activities to influence the transport of PF through BBB.

MDR1-transfected Madin-Darby canine kidney (MDCK-MDR1) cell, which is human P-gp-overexpressing, has some properties similar to the BBB. MDCK-MDR1 cell displays morphological enzymatic and antigenic cell markers, which are also found in cerebral endothelial cells, and have been reported as a suitable model *in vitro* for BBB [25,26]. MDCK-MDR1 cell was considered to be one of promising cells to determine the potential distribution of drugs in brain and the transport mechanism of drugs that passed the BBB [27,28]. The present study was undertaken to explore the transport mechanism of PF using MDCK-MDR1 cells modes *in vitro*, to evaluate the influence of LIG, SENI and SENA on the transport of PF, and to analyze the mechanism of the interactions.

## 2. Results

**Figure 2:**
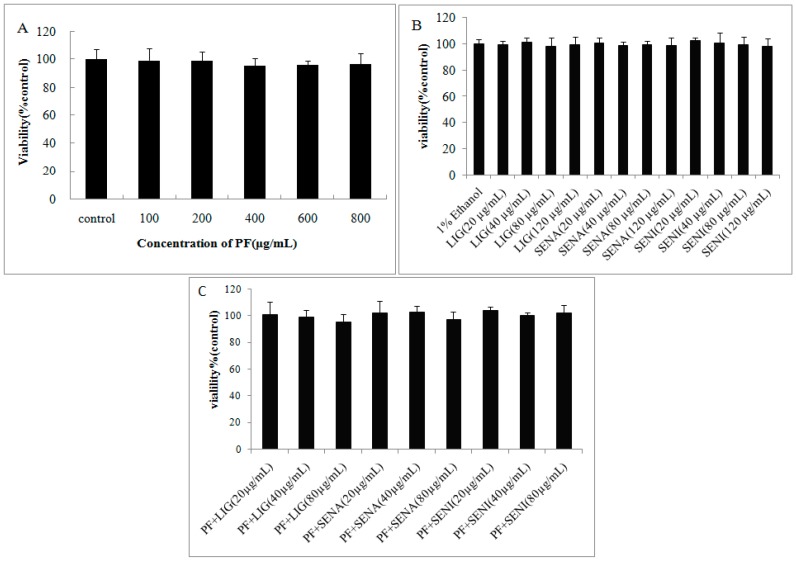
Cytotoxicity of paeoniflorin (PF) and its combination determined by 3-(4,5-dimethylthiazol-2-yl)-2,5-diphenyltetrazolium bromide (MTT) test on MDCK-MDR1 cells: (**A**) cytotoxicity of PF; (**B**) cytotoxicity of LIG, SENI and SENA; and (**C**) cytotoxicity of 400 µg/mL PF combined with LIG, SENI and SENA. Date are expressed as mean ±standard deviation (SD) (*n* = 6).

The cytotoxicity of PF was evaluated in MDCK-MDR1 cells with the aid of the MTT assay, and the results are shown in Figure 2. PF group has no cell cytotoxicity in the concentration range of 0–800 µg/mL. Therefore, we used concentrations within this range for subsequent experiments. LIG, SENA and SENI groups have no cell cytotoxicity in the concentration range of 0–120 µg/mL. The mixture of 400 µg/mL PF combined with three ingredients of chuanxiong (*w*/*w*) have no cell cytotoxicity in the concentration range of 0–80 µg/mL calculated with LIG, SENI, and SENA.

**Figure 3:**
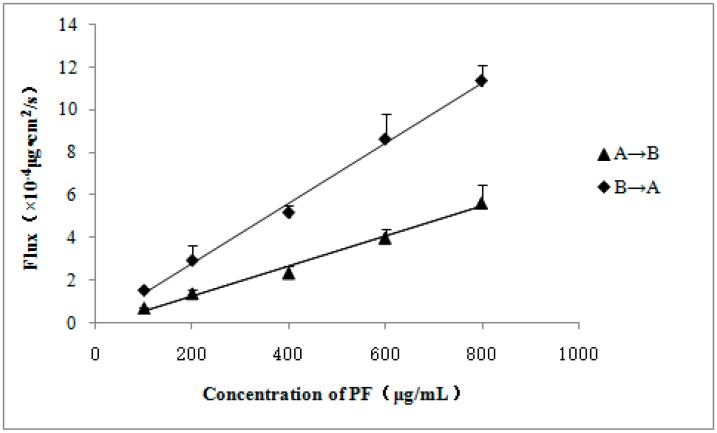
The Flux of PF transport across MDCK-MDR1 cell monolayers. A, apical side; B, basolateral side. Values are mean ± SD (*n* = 3).

Various concentrations of PF transports were tested across MDCK-MDR1 (Table 1). In general, drugs with high Papp (>1 × 10^−5^ cm/s) can be well-absorbed, while those with low Papp (<1 × 10^−6^ cm/s) are poorly absorbed [29]. The Papp (A→B) of PF between 0.587 and 0.705 × 10^−6^ cm/s in MDCK-MDR1 cells showed that it is relatively poorly absorbed. The Papp of PF in A→B direction did not differ significantly (Table 1), and the fluxes of PF were linearly associated with the concentration (Figure 3), which indicated that the transport of PF was concentration dependent, not saturable and mainly passive transport. The linear equation from apical side to basolateral side was y = 0.007x − 0.117 (*r* = 0.9945, *n* = 3), and the linear equation from basolateral side to apical side was y = 0.014x + 0.022 (*r* = 0.9975, *n* = 3), where y is the flux of PF and x is the concentration of PF. The B→A/A→B permeability *ER* of PF was more than 2 in the MDCK-MDR1 cells, which indicated that the transport mechanism of PF might be passive diffusion as the dominating process with the active transportation mediated mechanism involved.

To determine the potential role of P-gp in the transport of PF, a P-gp inhibitor, verapamil (VER), was added to medium that contained 400 μg/mL PF (Table 2). The Papp (A→B) of PF increased up to about 1.2-fold in comparison with the non-verapamil added (from 0.587 to 0.707 × 10^−6^ cm/s). In the presence of P-gp inhibitor, the B→A/A→B permeability ER of PF was 0.690, which suggested that PF was P-gp substance [30].

Although the surface area is much larger for the transcellular route, the paracellular route is the preferred absorption pathway for many water-soluble and poorly lipid-soluble drugs, ionized drugs, and high molecular weight compounds. EDTA-Na_2_ could destroy the intercellular structure that is named tight junction of the endotheliocyte and increase the paracellular permeation of hydrophilic macromolecules [31,32]. If the transport mechanism of a drug involves paracellular diffusion, its transport would improve when the tight junction structure is opened or destroyed. Thus, in this study, we investigated the transport of PF in the absence or presence of EDTA-Na_2_ (Table 3). The permeability of PF significantly increased when 2.5 mmol/L EDTA-Na_2_ was added (*p* < 0.01). The Papp (A→B) of PF increased up to about five-fold in comparison with the non- EDTA-Na_2_ added (from 0.587 to 2.736 × 10^−6^ cm/s). It suggested that PF was absorbed via the paracellular route. These results indicated that PF transported across the MDCK-MDR1 cell monolayers by both paracellular and transcellular diffusion.

**Figure 4:**
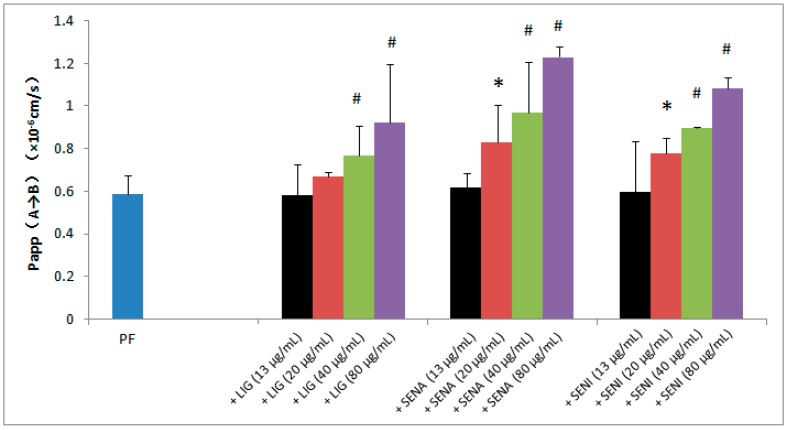
Effects of LIG, SENA and SENI on the transport of PF in MDCK-MDR1 cells. Papp, permeability; A, apical side; B, basolateral side. Values are mean ± SD (*n* = 3). Differs from PF (400 μg/mL): * *p* < 0.05, ^#^ *p* < 0.01.

The drug–drug interaction experiment of PF transport was carried out in MDCK-MDR1 cells to investigate the effect of bioactive ingredients of chuanxiong on its permeability. The changed transports of PF, caused by various concentrations of LIG, SENA and SENI, are shown in Figure 4. Results showed that transports of PF continuously increased in A→B direction with rising concentration of LIG, SENI and SENA. In the presence of LIG (40 and 80 µg/mL), the A→B fluxes of PF in MDCK-MDR1 were significantly increased (*p* < 0.01 and *p* < 0.05, respectively); in the presence of SENI or SENA (20, 40, and 80 µg/mL), the Papp (A→B) of PF in MDCK-MDR1 were significantly increased (*p* < 0.05, *p* < 0.01, and *p* < 0.01, respectively); and 80 µg/mL of SENA has the strongest promoting effect, for it significantly increased by approximately 109% the transport of PF in MDCK-MDR1 cell.

**Figure 5:**
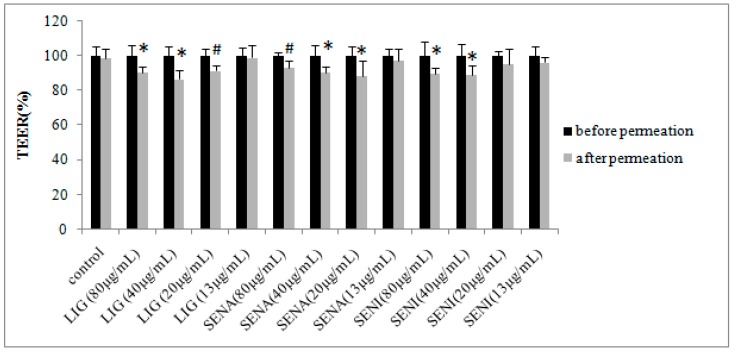
Transepithelial electrical resistance (TEER) before and after permeation of 400 μg/mL PF as control and in the presence of 400 μg/mL PF combined with LIG, SENI and SENA across MDCK-MDR1 monolayers. Data are expressed as mean ± SD (*n* = 3). * *p* < 0.05 and ^#^ *p* < 0.01 compared with the value before permeation.

Comparisons of the transepithelial electrical resistance (TEER) of the cell monolayer in the absence or presence of the three bioactive ingredients of chuanxiong are shown in Figure 5. TEER of cells treated with LIG (20 μg/mL) and SENA (80 μg/mL) significantly decreased during permeation studies (*p* < 0.01). TEER of cells treated with LIG (80 and 40 μg/mL), SENA (40 and 20 μg/mL) and SENI (80 μg/mL) markedly decreased during permeation studies (*p* < 0.05). A decrease in TEER during the permeation experiment can be explained by the modulation and opening of the tight junctions caused by the permeation enhancer LIG, SENI and SENA, but further research should be carried out to verify this.

**Figure 6:**
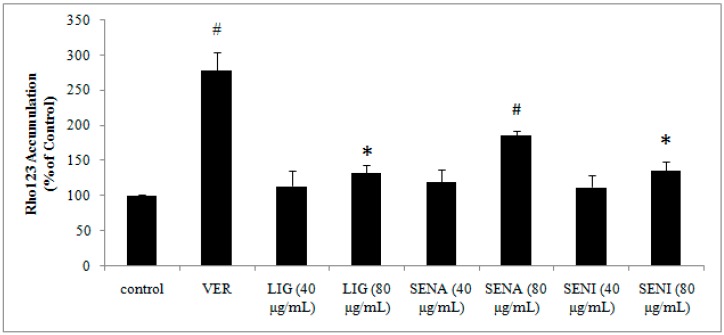
Effects of Ver, LIG, SENA and SENI on P-gp-mediated efflux function in MDCK-MDR1. Values are mean ± SD (*n* = 3). Differs from control group: * *p* < 0.05, ^#^ *p* < 0.01.

Rhodamine 123 (Rho 123) is a fluorescent dye that can be removed from the cells by P-gp function. Therefore, an increased cellular accumulation of Rho 123 is generally considered a marker of diminished P-gp efflux function [33]. The effects of different ingredients on P-gp-mediated efflux function in MDCK-MDR1 are shown in Figure 6. Verapamil, as P-gp inhibitor, significantly enhanced the intake of Rho 123 (*p* < 0.01). LIG, SENA and SENI could increase the intake of Rho 123 in a dose-dependent manner (Figure 5). Compared with control group, 80 μg/mL of SENA significantly increased by approximately 87% intake of Rho 123 (*p* < 0.01), while 80 μg/mL of LIG and SENI markedly increased by approximately 35% (*p* < 0.05). While 40 μg/mL of LIG, SENI and SENA increased by approximately 15% intake of Rho 123 when compared with control group, there was no significant difference (*p* > 0.05). These data suggested that LIG, SENI and SENA could down-regulate P-gp efflux function to enhance the transport of P-gp substrates, PF across BBB.

**Figure 7:**
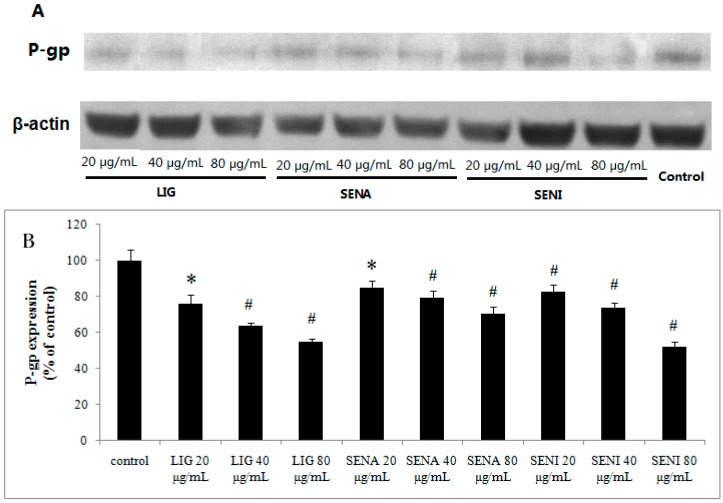
Effects of LIG, SENA and SENI on P-gp expression in MDCK-MDR1 cells: (**A**) representative P-gp expression by Western blot on MDCK-MDR1 treated with different concentration of LIG, SENA and SENI; and (**B**) quantitative data of P-gp expression. Values are mean ± SD (*n* = 3). Differs from control group: * *p* <0.05, ^#^ *p* < 0.01.

Western blot was used to investigate the effect of LIG, SENA and SENI on the expression of P-gp in MDCK-MDR1 cells. The results revealed a band of 170 kDa, corresponding to P-gp (Figure 7A). It was found that P-gp expressions in MDCK-MDR1 cells were markedly down-regulated by LIG, SENA and SENI. In Figure 7B, P-gp expressions of cells treated with LIG (20 μg/mL) and SENA (20 μg/mL) significantly down-regulated (*p* < 0.05). P-gp expressions of cells treated with LIG (40, 80 μg/mL), SENA (40, 80 μg/mL) and SENI (20, 40, 80 μg/mL) markedly down-regulated (*p* < 0.01). These data suggested that the enhanced transports of PF can be explained by the reduced expression of P-gp in MDCK-MDR1 cells caused by LIG, SENA and SENI.

Libdock (Discovery studio version 4.0) is commercial software for docking the ligands into active site of the protein. It calculates the hotspot map for the protein active site, which includes polar and apolar groups. This hotspot map is further used to align the ligands to form favorable interactions. Finally, it minimizes all the ligand poses and based on the ligands score they are ranked [34,35,36]. The docking analysis in Table 4 revealed the interaction of ligands-P-gp with the deep pocket of the identified binding site. The involved interactions between the small molecule and the pocket residues are numerous. Verapamil and PF with the highest LibDock Score indicated that they are P-gp substrate, which was consistent with the results in this experiment. Verapamil enhanced the transport of PF in MDCK-MDR1, and the mechanism was that verapamil could competitively bind with P-gp. LIG, SENA and SENI with lower LibDock Score can be explained by that they enhanced the transport of PF by inhibiting the expression of P-gp not by competitively binding with P-gp.

## 3. Materials and Methods

### 3.1. Materials

Polyester (PET) cell culture inserts and 12-well plates (12 mm diameter, 0.4 µm pore size) were purchased from Corning Costar Corporation (Cambridge, MA, USA). PF and verapamil were obtained from the National Institute for the Control of Pharmaceutical and Biological Products (Beijing, China). LIG, SENI and SENA were purchased from Chengdu Herbpurify Co., Ltd. (Chendu, China). Rhodamine 123 was purchased from Nanjing KeyGEN BioTeCH Co., Ltd. (Nanjing, China). Acetonitrile (Mreda Inc., Merck, Darmstadt, Germany) was of HPLC grade.

### 3.2. Cell Culture

MDCK-MDR1 cells were purchased fromCinoAsia co., Ltd. (Shanghai, China). Cells were cultured in Dulbecco’s modified Eagle’s media (DMEM, containing l-glutamineand 25 mM HEPES), supplemented with 10% heat-inactivated fetal bovine serum (FBS) as well as 100 U/mL penicillin and 100 µg/mL streptomycin. All cell lines were maintained at 37 °C with 5% CO_2_. The cells were seeded at a density of 250,000 cells/cm^2^, and were grown to confluence on PET inserts and maintained for seven days before the experiment. Fresh media were replaced to the cells every other day after seeding and 24 h before the transport study.

### 3.3. Cytotoxicity Assays

The capacity of PF, LIG, SENI and SENA to interfere with the growth of MDCK-MDR1 cells wa determined with the aid of the 3-(4,5-dimethylthiazol-2-yl)-2,5-diphenyltetrazolium bromide (MTT) dye assay. LIG, SENI and SENA were dissolved and diluted by HBSS containing 1% ethanol. Cells were seeded in 96 microtiter plates with flat-bottomed wells in a total volume of 200 µL of culture medium at a density of 1 × 10^5^ cells/mL. Plates were then incubated at 37 °C in a 5% CO_2_ atmosphere. After 24 h, the medium was removed and replaced with a fresh medium containing increasing concentrations of the compounds. After incubation for 24 h, 20 µL of a 5 mg/mL MTT solution in culture medium was added to each well and the mixtures were incubated at 37 °C until blue deposits were visible. The assay measures the amount of MTT reduction brought about by mitochondrial dehydrogenase and assumes that cell viability (corresponding to the reductive activity) is proportional to the production of purple formazan, which is measured spectrophotometrically. The colored metabolite was then dissolved in DMSO. Absorbance was measured at 490 nm with a Multiskan Go microplate reader (BioTek, Winooski, VT, USA). Wells with drugs and cells was used as sample group, and the absorption was recorded as A_sample_. Wells with the same concentration of drugs but without seeded cells were used as a blank control, and the absorption was recorded as A_blank_. Wells that contained cells but no sample solution were used as negative control, the absorption was recorded as A_control_. The mean absorbance of six measurements for each compound was expressed as a percentage of the absorbance of the untreated control and plotted against complex concentration.

### 3.4. Transport Studies Across MDCK-MDR1

Cell monolayers were pre-incubated in Hank’s buffered salt solution (HBSS, pH 7.4) for 30 min at 37 °C. Transepithelial electrical resistance (TEER) values were measured across the monolayers using a Millipore Millicell ERS system equipped with chopstick electrodes (Millipore Corporation, Billerica, MA, USA). Approximate MDCK-MDR1 values ranged from 500 to 800 Ω·cm^2^. To measure apical (A)→basolateral (B) transporter, 0.5 mL of drug solution was added in A side and 1.5 mL of HBSS in B side. Basolateral (B)→apical (A) transporter was evaluated adding 1.5 mL of drug solution in B side and 0.5 mL of HBSS in A side [37]. The final organic solvent concentration in HBSS was always kept below 1%, a concentration, which did not alter cell viability or permeability [38]. PF transport was studied at five concentrations: 100, 200, 400, 600 and 800 µg/mL. The effects of LIG, SENA and SENI on PF transport were studied at 13, 20, 40 and 80 µg/mL concentrations in the presence of 400 µg/mL PF. The P-gp inhibitor verapamil at 100 µmol/L concentrations was also used to study the changed transport of PF.

Each measurement was evaluated in triplicate. Cells were incubated in a 37 °C shaking incubator. Two hundred-microliter aliquots were taken from the apical side (to study B→A transport) and 500 µL of aliquots were taken from the basal side (to study A→B transport) at 30, 60, 90, 120, and 150 min time intervals. The same volume of fresh pre-warmed HBSS was added to keep the volume constant. 200 μL of PF samples was mixed with 180 μL methanol, and then 20 μL IS solution at 25.0 μg/mL was added, by vortex agitation for 3 min and then centrifuged for 10 min at 16,000 rpm. The supernatants were transferred to autosampler vials and injected into the LC-MS system.

### 3.5. LC-MS/MS Measurement of PF.

An HPLC system consisting of a solvent-delivery system LC-30 AD, an autosampler SIL-30 AC, a column oven CTO-30 AC, a solvent degasser DGU-20A3 and a controller CBM-20A from AB Sciex (Framingham, MA, USA) was used in the study. Separation was conducted using a Phenomenex Kinetex NB-C18 (100 × 3.0 mm, 2.6 μm, Torrance, CA, USA). The column oven was maintained at 30 °C. The mobile phase was water as mobile phase A and acetonitrile as mobile phase B. The linear gradient elution program was set according to preliminary tests: 5% B, 0–0.5 min; 5%–20% B, 0.5–2.0 min; 20%–40% B, 2.0–3.0 min; 40%–90% B, 3.0–5.0 min; 90% B, 5.0–8.0 min; 90-5% B, 8.0-8.1min; and 5% B, 8.1–12.0 min, with the flow rate kept at 0.4 mL/min. The injection volume was set at 5 μL.

The MS analysis was performed on a 4500 QTRAP™ system from Applied Biosystems (AB Sciex) equipped with Turbo V sources and Turbo Ionspray™ interface. Electrospray ionization was performed in negative mode. The mass spectrometric parameters were optimized as: Turbo Ion Spray (TIS) temperature, 650 °C; ion spray voltage, −4500 V; curtain gas, nitrogen, 30; nebulizing gas, 50; TIS gas, 50; declustering potential, −130 eV for PF, 0 eV for internal standard (IS, gastrodin); collision energy, −30 eV for PF, −35 eV for IS. The precursor-product ion pairs used in multiple reactions monitoring mode were 479.3–120.9 for PF, 285.0–122.8 for IS with dwell times of 100 ms; quadrupoles Q_1_ and Q_3_ were set on unit resolution. Analyst Software™ (version 1.6.1) was used to process the obtained data.

### 3.6. Data Analysis and Statistics

The percentage of cell toxicity in the MTT assay was calculated using the following Equation (1):

(1) $$\mathrm{Viability}(\%)\frac{A_{\text{sample}}-A_{\text{black}}}{A_{\text{control}}-A_{\text{black}}}\times 100$$

The apparent permeability coefficients (P_app_) for PF were calculated according to the following Equation (2), and the flux of PF was calculated according to Equation (3):

(2) $$P_{\text{app}}=\frac{\text{dQ}}{\text{dt}\times A\times C_{0}}$$

(3) $$\text{Flux}=\frac{\text{dQ}}{\text{dt}\times A}$$

where dQ/dt is the cumulative transport rate of the compound on the receiving side (mM/s), A is the surface area of the cell monolayer (cm^2^), and C_0_ is the initial concentration in the donor compartment (mM/cm). The efflux ratio (ER) was calculated according to the following Equation (4):

(4) $$\text{ER}=\frac{{\text{Papp}}_{\text{BL}\to \text{AP}}}{{\text{Papp}}_{\text{AP}\to \text{BL}}}$$

Experimentally derived *in vitro* data were shown as mean ± standard deviation (SD). Data were analyzed by one-way analysis of variance (ANOVA), followed by LSD test to analyze differences among multiple groups comparatively to control group (SPSS13.0 Statistical software). Significance was set at *p* < 0.05.

### 3.7. TEER Measurement

Transepithelial electrical resistance (TEER) of the monolayers was measured with the EVOM instrument (Millipore Corporation). The TEER of untreated cells and cells treated with LIG, SENI and SENA was determined in HBSS before and after the experiment. The measured TEER before the experiment was set as 100% and all other values were calculated according to this.

### 3.8. The Effects of LIG, SENI and SENA on Rhodamine 123 Accumulation

Rho 123 efflux assay was used to measure the activity of P-gp in MDCK-MDR1 cells according to previous methods [39]. In total, 5 × 10^5^ cells (1 mL) grown to confluency in a heparinized eppendorf (EP) tube were treated with 20, 40 and 80 µg/mL of LIG, SENI, or SENA, or with 100 µmol/L verapamil, for 1 h, and subsequently incubated in 5 µmol/L rhodamine123 for 1 h. After incubation with rhodamine 123, cells were washed with ice-cold HBSS for three times and solubilized in 0.5 mL *n*-butyl alcohol. Fluorescence of rhodamine 123 was measured with emission wavelength at 535 nm and excitation wavelength at 485 nm using a fluorescence spectrophotometer (Victor, PerkinElmer, Waltham, MA, USA). Each experiment was repeated at least three times to ensure uptake and consistency and reproducibility of the experiments.

### 3.9. Western-Blot Analysis

Cells treated with different concentrations of LIG, SENA and SENI for 8 hours were subject to Western blot assay. Proteins were extracted from whole cell lysates and separated by sodium dodecyl sulfate–polyacrylamide gel electrophoresis, then transferred to a polyvinylidene fluoride (PVDF) membrane. The following primary antibodies were used: rabbit anti-P-gp (1:1000; Abcam, Cambridge, MA, USA) and mouse anti-β-actin (1:2000; Abcam). Membranes were then incubated with the horseradish peroxidase-conjugated secondary anti-bodies (1:5000; Abcam). The transferred proteins were incubated with enhanced chemiluminescence (ECL) substrate solution and visualized with the Image J program (Bio-Rad, Richmond, CA, USA). The relative levels were quantified densitometrically by using the Quantity One software (Bio-Rad Laboratories) and calculated according to the reference bands of β-actin.

### 3.10. Molecular Docking

The ligands were PF, LIG, SENI, SENA and verapamil .The molecular structure of the ligands was found from NCBI [40] and the SDF file format used for molecular docking was downloaded. The X-ray crystal structure of P-gp (PDB code: 3G60) was retrieved from RCSB Protein Data Bank (4.40 resolution) [41]. This complex structure consists of two homodimeric chains, A and B. Discovery Studio 4.0 (DS) was employed to perform the docking of the compounds into the active sites of P-gp. The protein was prepared by removing crystal water and other hetero atoms, followed by addition of hydrogen, protonation, ionization and energy minimization. The CHARMm force field was applied for geometry optimization. Met68, leu300, tyr303, phe332, leu335, ile336, phe339, gln721, phe724, phe728, leu758, phe833, tyr949, phe974, ser975, val978, ala981, met982, gly985, gln986, and ser989 were amino acid residues of P-gp, and were defined as active sites in the studies [42]. The detailed binding modes of ligands with P-gp were illustrated by docking all the prepared ligands at the defined active site using Libdock, and the Libdock scores were recorded for analysis [43,44].

## 4. Conclusions

In conclusion, PF showed relatively poor absorption in the BBB models. The bidirectional transport of PF across MDCK-MDR1 monolayers was concentration dependent and not saturable. The B→A/A→B permeability *ER* of PF was more than 2 in the MDCK-MDR1 cells, which indicated that the transport mechanism of PF might be passive diffusion as the dominating process with the active transportation mediated mechanism involved. The increased Papp of PF in A→B direction by EDTA-Na_2_ suggested that PF was absorbed via the paracellular route. The B→A/A→B permeability *ER* of PF decreased from 2.210 to 0.690 in the presence of verapamil, which suggested that PF was a substance of P-gp. LIG, SENA and SENI could increase the Papp of PF in A→B directions in a concentration-dependent manner. LIG, SENA and SENI behave as effective absorption enhancers in BBB by reducing the expression of P-gp and opening tight junctions. Since there are other proteins to cover the whole functional features of the BBB, there are some limitations of the findings and further experiments about the enhance mechanism of chuanxiong are expected.

## Figures and Tables

**Table 1:** Increasing concentration of PF transport across MDCK-MDR1 cell monolayers.

Concentration of PF (μg/mL)	Papp (A→B) (×10^−6^ cm/s)	Papp (B→A) (×10^−6^ cm/s)	ER (B→A/A→B)
100	0.697 ± 0.053	1.549 ± 0.092	2.222
200	0.691 ± 0.082	1.475 ± 0.363	2.134
400	0.587 ± 0.088	1.297 ± 0.084	2.210
600	0.666 ± 0.072	1.436 ± 0.201	2.156
800	0.705 ± 0.107	1.418 ± 0.097	2.011

Papp, permeability; A, apical side; B, basolateral side. Values are mean ± SD (*n* = 3).

**Table 2:** The effect of P-gp inhibitors on transport of PF in MDCK-MDR1 cell monolayer.

Group	Papp (A→B) (×10^−6^ cm/s)	Papp (B→A) (×10^−6^ cm/s)	ER (B→A/A→B)
400 μg/mL PF	0.587 ± 0.088	1.297 ± 0.084	2.210
400 μg/mL PF + 100 mmol/L verapamil	0.707 ± 0.062 *	0.488 ± 0.093 ^#^	0.690

Papp, permeability; A, apical side; B, basolateral side. Values are mean ± SD (*n* = 3). Differs from PF (400 μg/mL): * *p* < 0.05, ^#^ *p* < 0.01.

**Table 3:** The effect of EDTA-Na_2_ on transport of PF in MDCK-MDR1 cell monolayer.

Group	Papp (A→B) (×10^−6^ cm/s)
400 μg/mL PF	0.587 ± 0.088
400 μg/mL PF + 2.5 mmol/LEDTA-Na_2_	2.736 ± 0.212 ^#^

Papp, permeability; A, apical side; B, basolateral side. Values are mean ± SD (*n* = 3). Differs from PF (400 μg/mL): ^#^ *p* < 0.01.

**Table 4:** The detailed binding modes of ligands with P-gp and the LibDock Socre.

NO	English Name	The Type of Interaction	The Key Amino Acids	LibDock Score
1	PF	hydrogen-bonding interaction	gln721 and ser 725	139
		hydrophobic interaction	val978, leu971, and phe724	
2	LIG	hydrogen-bonding interaction	ser 975	86
		hydrophobic interaction	phe974, phe332, phe724, val978, and phe728	
3	SENI	hydrogen-bonding interaction	ser725	91
		hydrophobic interaction	leu971, phe728, phe332, phe724, and val978	
4	SENA	hydrogen-bonding interaction	ser729	89
		hydrophobic interaction	phe71, phe974, phe332, phe728, val978	
5	Verapamil	hydrogen-bonding interaction	gln721	138
		hydrophobic interaction	tyr303, tyr306, leu335, phe332, phe724, met 982, val978, and phe728	
